# GENESIS – The GENEric SImulation System for Modelling State Transitions

**DOI:** 10.5334/jors.179

**Published:** 2017-09-20

**Authors:** Matthew S. Gillman

**Affiliations:** Wolfson Institute, Queen Mary University of London, UK

**Keywords:** simulation, modelling, disease progression, state transitions, state machine, probabilities, Perl, C++, random number generation, Markov chain, Markov process

## Abstract

This software implements a discrete time Markov chain model, used to model transitions between states when the transition probabilities are known *a priori*. It is highly configurable; the user supplies two text files, a “state transition table” and a “config file”, to the Perl script genesis.pl. Given the content of these files, the script generates a set of C++ classes based on the State design pattern, and a main program, which can then be compiled and run. The C++ code generated is based on the specification in the text files. Both multiple branching and bi-directional transitions are allowed.

The software has been used to model the natural histories of colorectal cancer in Mexico. Although written primarily to model such disease processes, it can be used in any process which depends on discrete states with known transition probabilities between those states. One suitable area may be in environmental modelling.

A test suite is supplied with the distribution.

Due to its high degree of configurability and flexibility, this software has good re-use potential. It is stored on the Figshare repository.

## (1) Overview

### Introduction

Software simulations cover a huge field, ranging from agent models to differential equation-based systems with an underlying physical model. One class of models which can be simulated are known as Markov models. These are models which have a discrete set of states which can be moved between with defined probabilities; each state has no knowledge of the preceding state(s) encountered. Markov models have recently been used to model prevention and treatment of the hepatitis B virus [1], bacteria secretion [2], protein cell binding [3] and the lengths of tree branches [4].

The majority of published simulation models have been developed to address a specific disease or process, like those available at CISNET [5]. Genesis, however, is a **generic discrete-time Markov chain** simulator. It models a series of states with specified transition probabilities between those states. It does not have the specific functionality of a more specialised simulator.

Genesis is written in Perl and C++. Other authors have written similar software in other programming languages; for example, packages markovchain [6] and DTMCPack [7] are written in R.

Genesis developed from a microsimulation model written for the investigation of cervical cancer screening by Landy *et al* [8]. However, like the CISNET models, the model used in Landy *et al* is optimised for a specific disease and different screening scenarios.

The software has been used initially to look at the natural histories of colorectal cancer in Mexico, which were then used to compare the potential impact of screening programmes [9].

Although this paper describes the software, more detailed instructions can be found in the README.txt file supplied with it.

### Implementation and architecture

**Figure 1:**
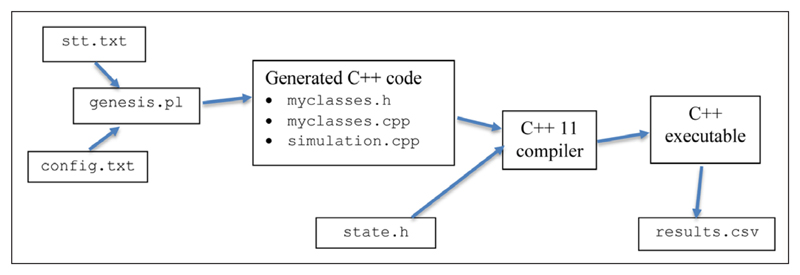
Steps to generate the software and results.

Figure 1 presents an overview of how the software works and, ultimately, generates results. After downloading from the repository, the steps to run the software are as follows:

1. Create a suitable “state transition table” file, stt.txt, and configuration file, config.txt. Initially the examples supplied can be used.
2. Run the genesis.pl Perl script. This, and the accompanying Perl module GenesisFunc.pm, will generate C++ files (myclasses.h, myclasses.cpp and the client simulation.cpp) with classes based on the State design pattern, using the information supplied in the .txt files.
  Usage example (all platforms):
  perl genesis.pl
3. Compile the C++ code into the simulation executable.
  Usage example (Linux) (the following should all be on one line):
  g++ -std=c++11 -std=gnu++11
    simulation.cpp myclasses.cpp -o simulation
  Usage example (Windows):
  cl /EHsc simulation.cpp myclasses.cpp
4. Run this executable, which creates a file called results.csv.
  Usage example (Linux):
  ./simulation
  Usage example (Windows)
  simulation
  Note that helpful output is seen as the program executes. However, for larger simulations this can be copious and slow down execution, in which case it may be desirable to send this output to a null device.
  Usage example (Linux):
  ./simulation > /dev/null
  Usage example (Windows):
  simulation > NUL
5. Finally, the results.csv file can be examined and used to extract the required information.

During the development phase, the software was initially coded in C++, using and adapting the example implementation of the State pattern given on the Sourcemaking website [10]. Once this was accomplished, the genesis.pl script was written to automatically generate the C++ code expected from stt.txt and config.txt.

Each state mentioned in stt.txt will result in a C++ class being generated. These are children of the abstract base class State (declared in state.h). They implement the goNext() function which determines which state the program should progress to next (including staying in the same state).

The example stt.txt file in the code repository has the following lines (the line ordering is irrelevant):

occult symp 12 20 0.05
occult symp 20 40 0.06
occult treated 12 80 0.07
normal cin1 12 80 0.2
cin1 occult 12 80 0.3

**Figure 2:**
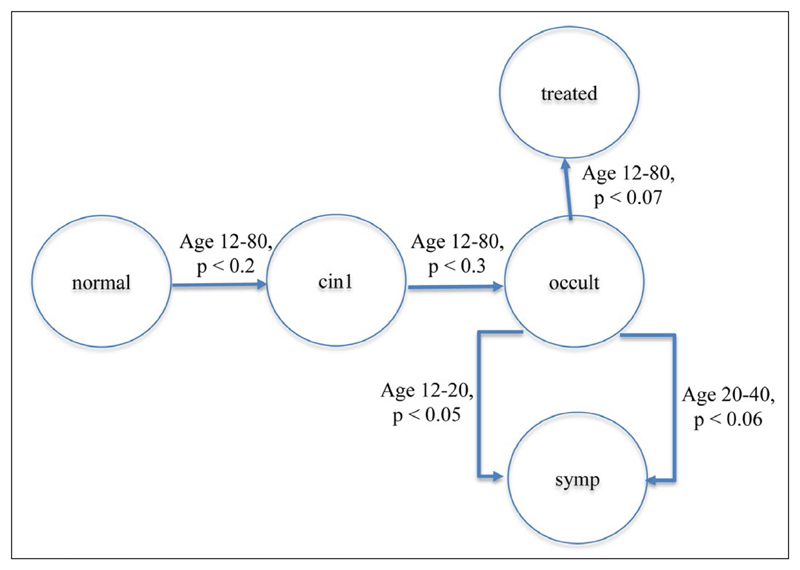
State diagram based on the example text files.

Each line consists of a source state name, sink state name, minimum age, maximum age and finally probability. Here, possible states are *occult, symp, treated, normal* and *cin1*. The first line means “if the person is currently in the *occult* state, and aged 12 or more and less than 20, there is a probability of 0.05 per time interval that they will transition to the *symp* state”. *Occult* is a “source” state and *symp* a “sink” or “absorbing” state. The state diagram for this example is shown in Figure 2. Not shown is the fact that a subject can “transition” from one state to itself, i.e. they can remain in that state. For an environmental process, the 12 and 20 might refer to, for example, the height of sea level or a distance.

Note that if the time interval used is changed, e.g. from years to months, the ages and probabilities will have to be adjusted appropriately.

The final states in this example are *treated* and *symp*. This is a particularly simple example as it is uni-directional; lines could be added to the stt.txt file to model the probabilities of leaving these states. Given two states *A* and *B*, it is possible to define transition probabilities both for *A* to *B* and *B* to *A*. In such a case *A* and *B* would each be both source and sink states.

Note that the model states generated by this software have the Markov property; a state does not have knowledge of the history the individual had prior to entering that state. This may be undesirable for some simulation scenarios; one solution might be, for example, rather than moving from a cancerous state back to a *normal* state, moving instead to an *in_remission* state.

**Figure 3:**
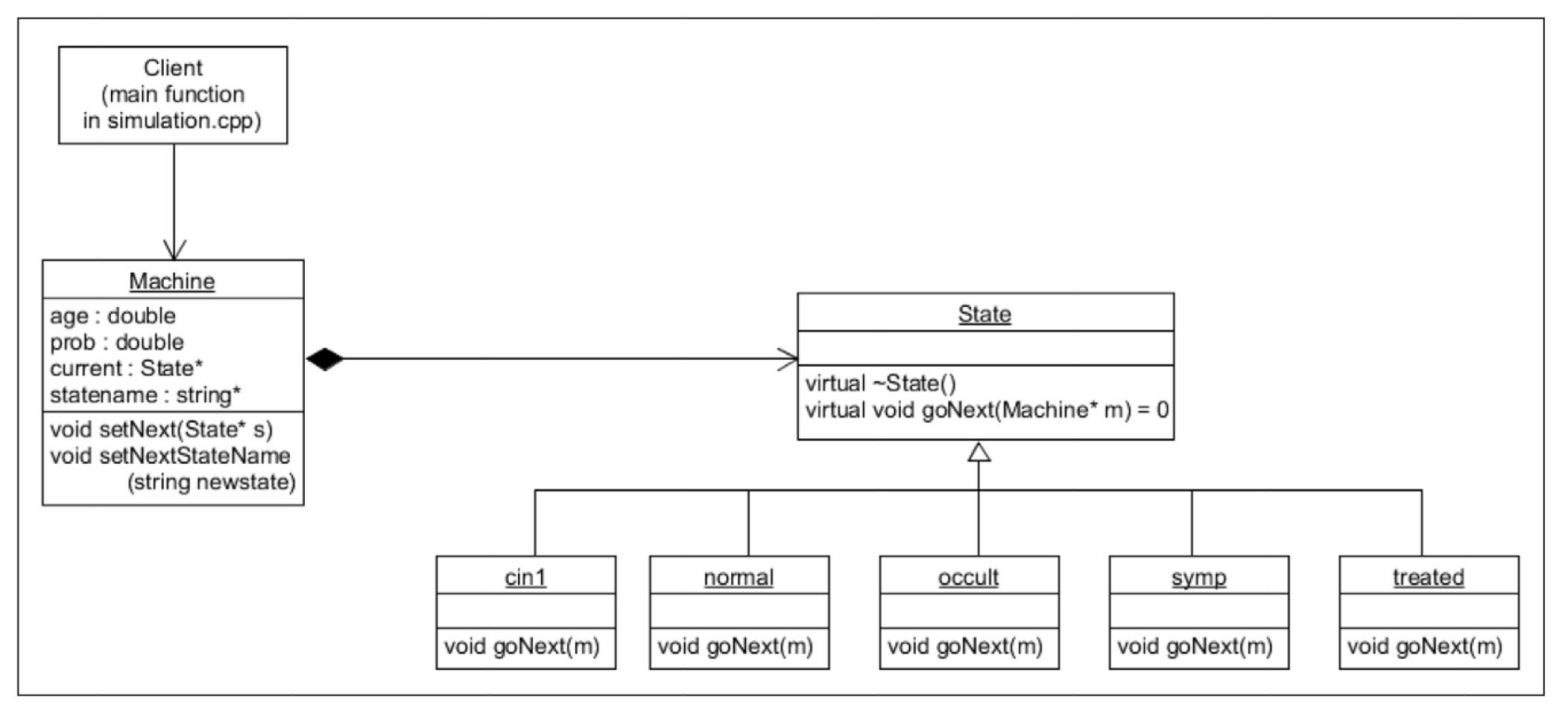
UML class diagram for the example.

A class diagram of the C++ code produced by this example is shown in Figure 3. The Machine class is the means by which client code can connect to the state machine.

For this example, the goNext() function for the *occult* state/class will have C++ code generated similar to the following pseudocode. R is a number (between 0 and 1) from the random number generator; a new value for R is generated at each age step of the model.

if (age is between 12 and 20) {

        if (R < 0.05) go to *symp* state

        else if (R > = 0.05 and R < (0.05 + 0.07)) go to *treated*

        state

        else stay in *occult* state (with implicit probability

        1–(0.05 + 0.07) = 0.88)

}

else if (age is between 20 and 40) {

        if (R < 0.06) go to *symp* state

        else if (R > = 0.06 and R < (0.06 + 0.07)) go to *treated*

        state

        else stay in *occult* state (with implicit probability

        1–(0.06 + 0.07) = 0.87)

}

else if (age is between 40 and 80) {

        if (R < 0.07) go to *treated* state

        else stay in *occult* state (with implicit probability

        1–0.07 = 0.93)

}

else stay in *occult* state (all other ages)

In this example, note the implicit probabilities of staying in the *occult* state. If necessary these can be explicitly stated in the stt.txt file. For example, the first implicit probability listed above is 0.88; this could be given in stt.txt as:

occult occult 12 20 0.88

If this is done (a) it gives confidence that the correct probability for the model to remain in that state has been specified and (b) for a given source state and age range, the software checks that the total of the transition probabilities (including from a state to itself) defined in stt.txt do not exceed a total of 1.

The config.txt file for this example has the content:

startage:12
stopage:80
interval:2
number:5

More details of config.txt, and the default values used if not specified in config.txt, are given in the README.txt file supplied with the distribution. In particular it is possible to specify the same seed to start the random number generator (a default is used here as it is not specified in the example above), or the user’s system clock can be used to generate a “random” seed.

Here, the initial age to simulate is 12 time units (each time unit is one year in this example), in steps of 2 time units up to 80. Five individuals will be simulated, one after the other. The random number generator is not reset between individuals.

As the initial state has not been otherwise specified, the simulation starts in the default initial state (*normal*). In order to start in a different state, e.g. *cin1*, the following line would be needed in config.txt:

initialstate:cin1

The desired state to start the simulation in must correspond to a source state in stt.txt.

To generate probabilities between 0 and 1, the standard C++ Mersenne Twister engine is used to sample from a uniform distribution. If, say, a transition probability from state *A* to state *B* of 0.2 was specified in stt.txt, that particular transition would occur only if the next random number in the sequence was from 0 (inclusive) to just less than 0.2. If, additionally, a probability of 0.15 was defined for the transition state *A* to state *C*, then this would occur if the random number was from 0.2 (inclusive) to just less than 0.35 (=0.2 + 0.15). (The software may not follow this exact ordering).

The output from running the C++ simulation executable is a comma-separated value file, results.csv. This has a header row starting with “ID” (for *i*^th^ simulation) and then a sequence of numbers (e.g. denoting different ages). The data lines follow. Examples are given in the next section.

### Quality control

The software has been functionally tested on Linux and Windows. On Linux, it was compiled with a number of options:

g++ -g3 -Wall -Wextra -Wstrict-overflow=5
    -std=c++11 -Wno-unused-parameter
    -ansi -pedantic -W -Wconversion
    -Wshadow -Wcast-qual -Wwrite-strings
    -std=gnu++11 simulation.cpp myclasses.cpp
    -o simulation

To identify and remove problems such as pointer errors and memory leaks, it was run under Valgrind.

A test suite has been written to test the core Genesis functionality housed in GenesisFunc.pm. The test script, genesis.t, should be used with the supplied files test.config.txt and test.stt.txt. All tests should pass. The script may be run with the command:

perl genesis.t

If any tests fail, a message will state this fact at the end of the standard output produced.

The file output from the compiled simulation executable is called results.csv. On Windows this can be opened in Excel and the columns of interest examined, e.g. the number of people in each state at a given age. On Linux it is possible to use command-line utilities to do the same, e.g.

cut -f 34 -d ‘,’ results.csv |sort| uniq –c

will show how many people are in each state at the age (or stage) corresponding to the 34^th^ comma-separated column.

It is possible to run a fast test to ensure that the software is working as planned. Suppose there exists a simple two-state model, with one thousand people, all of whom start in state *healthy*, and with a 50% chance per time interval that they will transition to state *cin1*. Initially the model is run for only one time interval. Then stt.txt might be:

normal cin1 12 80 0.5

and config.txt:

startage:20
stopage:21
interval:1
number:1000

Running through the Perl script/C++ compilation/C++ invocation process should then produce content similar to:

ID,20,21,
1,normal,normal,
2,normal,cin1,
3,normal,cin1,
4,normal,cin1,
5,normal,normal,
...
1000,normal,normal

The first column is the “person” ID. Every person at age 20 will be in the initial, *normal* state (this can be checked for safety).

On Windows, the resulting columns can be examined in Excel. On Linux, it is possible to find the number of people in each state at age 21:

$ cut -d ‘,’ -f3 results.csv |sort|uniq -c
      1 21
    523 cin1
    477 normal

showing that 52.3% of people transitioned into the *cin1* state.

If the number of people simulated is then increased to one million, the config.txt file will be as above except for the line

number:1000000

Again running the Perl script, and recompiling and running the C++ executable, might produce results similar to:

$ cut -d ‘,’ -f3 results.csv |sort|uniq -c
      1 21
  50146 cin1
  49854 normal

showing that about 50.1% of people transitioned to *cin1*. Thus, as the number of people simulated is increased, the limiting case of 50% is approached.

This experiment can be extended by changing the relevant line in config.txt to

stopage:22

such that two intervals (each of length 1 unit) are considered. Typical results might be:

ID,20,21,22,
1,normal,normal,cin1,
2,normal,normal,cin1,
3,normal,cin1,cin1,
4,normal,normal,normal
...

In this case the results after one interval (i.e. at age 21) will be similar to those above, but those after two intervals (age 22) will tend towards 75% in *cin1*.

## (2) Availability

### Operating system

Linux, Windows

### Programming language

Perl and C++ 11.

### Additional system requirements

Minimal disk space and memory are required to run this software.

### Dependencies

Standard C++ library required.

### List of contributors

Matthew Gillman. ORCID ID: 0000-0002-2340-6930.

### Software location

***Archive*** 

***Name:*** GENESIS – the GENEric SImulation System for modelling state transitions.
  ***Persistent identifier:*** DOI: https://doi.org/10.6084/m9.figshare.4775437
  ***Licence:*** MIT
  ***Publisher:*** Matthew Gillman
  ***Version published:*** 1.0
  ***Date published:*** 18/05/17

***Code repository*** 

***Name:*** N/A
  ***Identifier:*** N/A
  ***Licence:*** N/A
  ***Date published:*** N/A

***Emulation environment (if appropriate)*** 

***Name:*** N/A
  ***Identifier:*** N/A
  ***Licence:*** N/A
  ***Date published:*** N/A

### Language

English

## (3) Reuse potential

This software was envisaged initially as being used to model disease progression, but it can be used for any situation or field where a discrete set of states exist with defined transition probabilities between those states. Thus it may be possible to model processes from a wide range of fields.

It has been used to model the natural history of colorectal cancer in Mexico. Given known transition probabilities from the literature, a cohort of 1 million individuals was simulated from a given starting age, and their natural histories followed over time. Thus the simulation estimated the number of cancers (including occult (hidden) ones) which would be expected at each time point, assuming there was no medical intervention. With knowledge of the test sensitivity of screening methods, it was then possible to estimate how many of the occult cancers would be detected by screening, and hence how many clinical cancers (and potentially deaths) could be avoided by such screening.

Caution should be used when using config.txt and stt.txt files on Linux supplied from Windows, e.g. from another user. It is best to run such files through the Linux utility dos2unix before running genesis.pl and then compiling. This will prevent Windows ^M metacharacters appearing in simulation.cpp. Note that this is not necessary with the files supplied in the distribution.

For limited support, please contact the author via the email address given on his ORCID page (http://orcid.org/0000-0002-2340-6930).
